# Escherichia coli-Induced Psoas Abscess in a Case of Chronic Kidney Disease

**DOI:** 10.7759/cureus.28195

**Published:** 2022-08-20

**Authors:** Vijay Kota, Siva Reddy, Ruchita Kabra, Sourya Acharya, Sunil Kumar

**Affiliations:** 1 Department of Medicine, Jawaharlal Nehru Medical College, Datta Meghe Institute of Medical Sciences, Wardha, IND; 2 Internal Medicine, Datta Meghe Institute of Medical Sciences, Wardha, IND

**Keywords:** e-coli, immunocompromised, spine, infection, hematogenous

## Abstract

A psoas abscess is an accumulation of pus in the muscular compartment of the iliopsoas. It can originate from a primary or secondary source. Hematogenous or lymphatic seeding from a distant place causes primary iliopsoas abscess. This is frequently linked to a chronic immunocompromised status and is more common in young people. Secondary psoas abscess is caused by infection spreading directly from a nearby structure to the psoas muscle, and it can be caused by trauma or instrumentation in the inguinal region, lumbar spine region, and hip region. Occurrence of psoas abscess is uncommon, and its diagnosis is frequently delayed due to non-specific symptoms.

We discuss a patient with chronic kidney disease (CKD) on conservative management who presented to us with complaints of swelling in the right lower back and fever and who was subsequently diagnosed with a right psoas abscess. Microbiology culture of the pus confirmed *Escherichia coli (E. coli)* as the etiologic agent, which is rare.

## Introduction

A psoas abscess is an accumulation of pus in the psoas muscle, which is anatomically situated between the twelfth thoracic and fifth lumbar vertebrae. The psoas muscle arises from the tip of the transverse processes and the lateral surfaces of the vertebral bodies, and then extends inferiorly across the brim of the pelvis, below the inguinal ligament, creating a tendon with the iliacus, and into the lower trochanter of the femur, which is the hip joint's flexor.

Psoas abscess usually occurs in immunocompromised states like diabetes mellitus (DM), chronic renal failure (CRF), AIDS, or IV drug use. It usually affects children and young adults. Over 88% of individuals with primary iliopsoas abscess have *Staphylococcus aureus* as the causative pathogen. *Streptococcus* species cause 4.9% of secondary iliopsoas abscesses, and *Escherichia coli (E. coli)* causes 2.8% [1].

The incidence of psoas abscess in India is around 0.4/100,000, with a male-to-female ratio of 1.62:1 [2]. The mortality rate of iliopsoas abscess has been reported to be as high as 19% [3]. Fever, flank pain, loss of weight and appetite, lump in the flank, and decreased range of motion of the hip joint are all signs of an iliopsoas abscess. Diagnosis of iliopsoas abscess is frequently delayed due to non-specific presenting symptoms. The treatment of psoas abscess depends on managing pre-existing medical issues and ensuring surgical readiness. Drainage and a quick introduction of suitable antibiotics are the mainstays of iliopsoas abscess treatment. The majority of abscesses are small in size and can be successfully treated with antibiotics alone and those that require drainage can be successfully treated with imaging modalities such as USG- or CT-guided drainage. Antibiotic treatment should be adjusted based on the identified causative organisms.

## Case presentation

A 72-year-old male, a known case of chronic kidney disease (CKD) on conservative management, presented to us with complaints of fever and swelling in the right loin for 15 days and decreased appetite of five-day duration. The patient had no history of cough, expectoration, breathlessness, burning micturition, or haematuria.

A general examination showed blood pressure of 110/70 mmHg, and a pulse of 120/minute, regular. Other system examinations were normal. The local examination revealed a firm swelling of 6.5 x 9.5 cm in size, which was oval in shape, in the right flank, which was tender to touch. The local temperature was elevated. Consistency was soft to firm with positive fluctuation. Transillumination was negative.

A summary of the investigations performed on the patient is presented in Table 1.

**Table 1:** Investigations on the patient at the time of admission

Investigations	Patient value	Normal range
Hemoglobin	9 gm%	12-14 gm%
White blood cells	18,500 cu/mm	6,000-11,000 cu/mm
Platelet counts	2.49 lakh/mm	1.5-4.5 lakh/mm
Urea	180 mg/dl	9-20 mg/dl
Creatinine	3.5 mg/dl	0.6-1.2 mg/dl
Sodium	133 mmol/L	135-145 mmol/L
Potassium	4.3 mmol/L	3.5-5.5 mmol/L
Alkaline phosphatase	345 U/L	38-126 U/L
Alanine transaminase	9 U/L	<50 U/L
Aspartate aminotransferase	16 U/L	17-59 U/L
Total proteins	5.8 gm/dl	6.3-8.2 gm/dl
Albumin	2.4 gm/dl	3.5-5 gm/dl
Total bilirubin	0.4 mg/dl	0.2-1.3 mg/dl
Magnesium	2 mg/dl	1.6-2.3 mg/dl
Phosphorus	4.7 mg/dl	2.5-4.5 mg/dl
Uric acid	11.6 mg/dl	3.5-8.5 mg/dl

**Figure 1:**
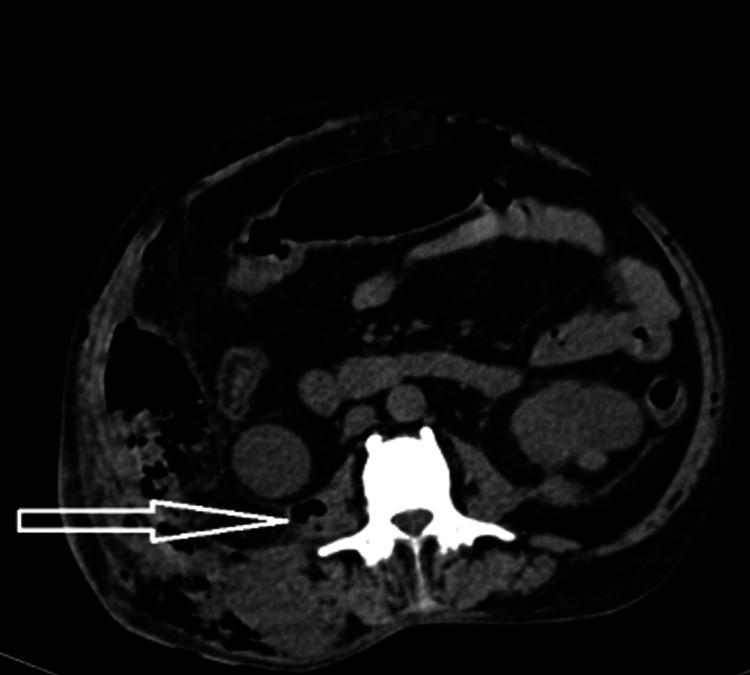
Contrast-enhanced CT abdomen axial section showing a collection and air focus along the right psoas muscle CT: computed tomography

**Figure 2:**
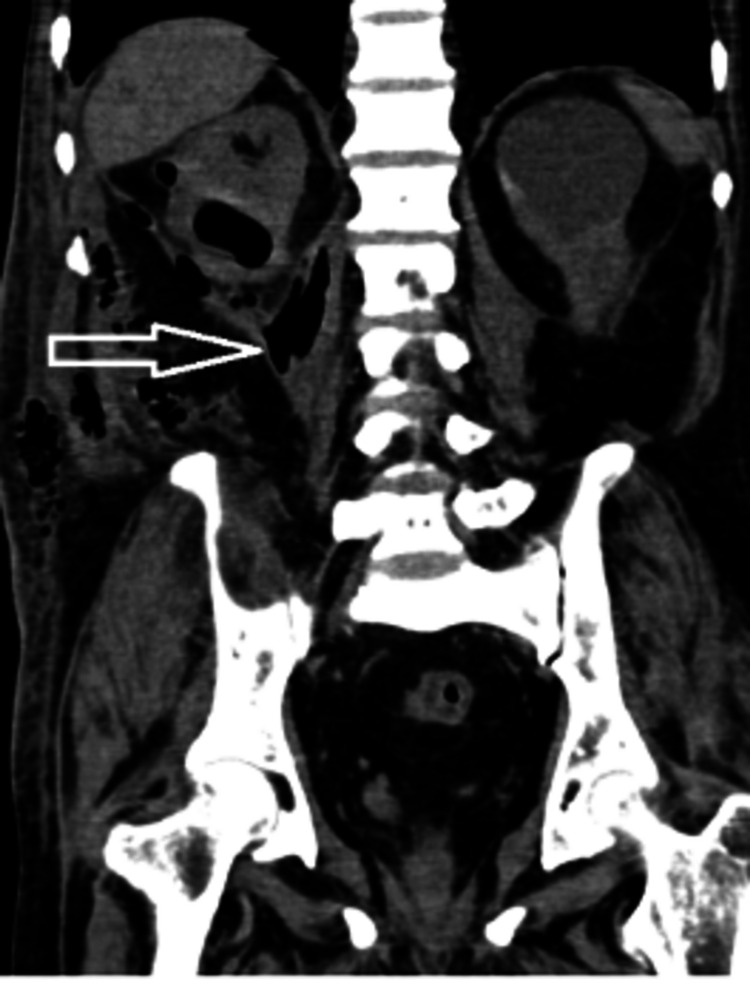
Contrast-enhanced CT abdomen coronal section showing a collection and air focus along the right psoas muscle CT: computed tomography

Triphasic CT of the abdomen and pelvis revealed a heterogeneously dense collection involving the right psoas muscle extending to the right perinephric space anterolaterally and to the right paraspinal muscles. The collection was breaching the right lateroconal fascia and was extending to the extraperitoneal space along the right lateral abdominal wall, reaching up to the right iliac fossa. Multiple air pockets along the extraperitoneal space of the abdomen extended to the space of Retzius. Collection along the right psoas muscle measured 6.5 x 5.5 x 9.5 cm as shown in Figures 1-2.

**Figure 3:**
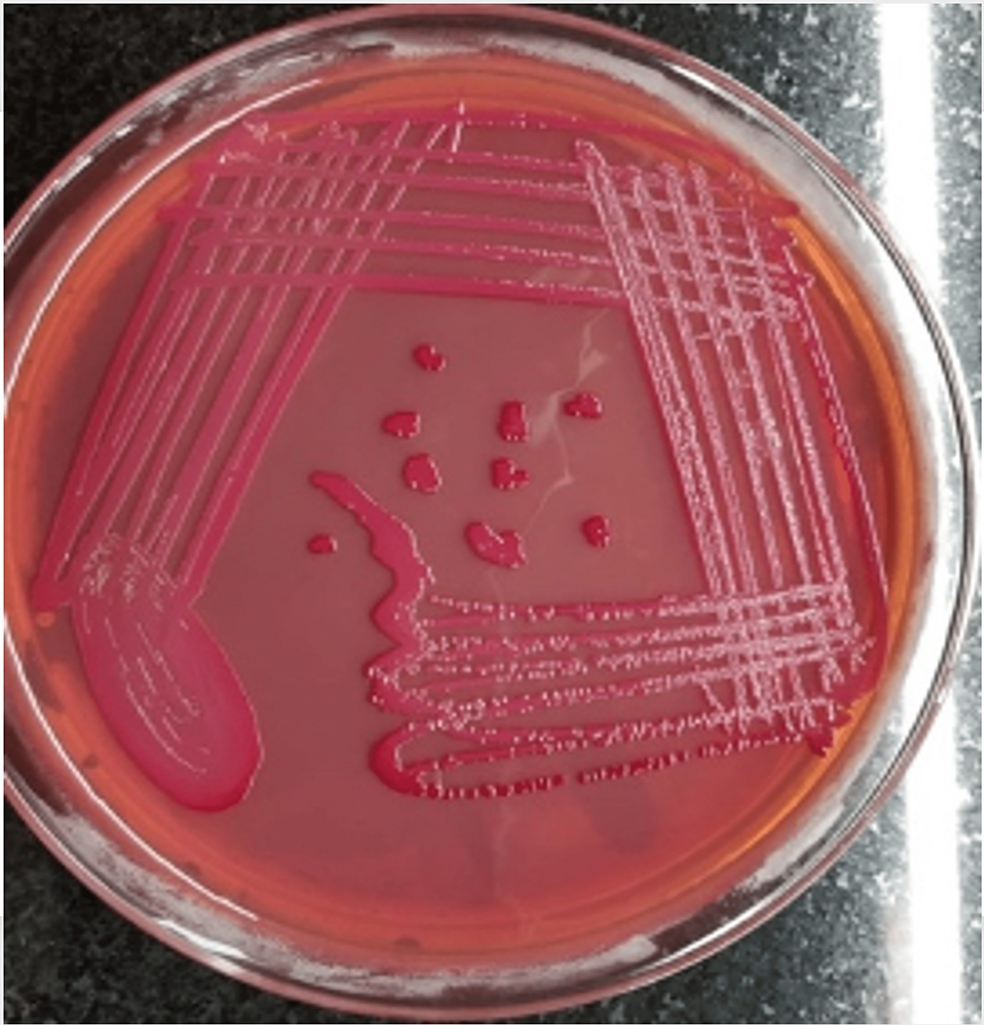
Growth of E. coli in blood agar E. coli: Escherichia coli

A pigtail drainage was done, and around 450 ml of pus was drained. The culture of the aspirated pus revealed the growth of *E. coli* as shown in Figure 3, which was sensitive to aztreonam and meropenem. Pus for cartridge-based nucleic acid amplification test (CBNAAT) was negative for *Mycobacterium tuberculosis*. The patient was started on injectable meropenem 500 mg IV BD and, later on, incision and drainage were done in view of the spreading of pus in superficial layers, and around 200 ml of pus was drained; the patient was discharged in a healing state after two weeks of antibiotic therapy.

## Discussion

The mortality rate of iliopsoas abscess has been reported to be as high as 19%. Fever, flank pain, loss of appetite and weight, lump in the right flank, decreased range of motion of the hip joint, and also laboratory findings of leukocytosis, elevated C-reactive protein (CRP), and high erythrocyte sedimentation rate (ESR) are all indications of an iliopsoas abscess. Diagnosis of psoas abscess is frequently delayed due to non-specific presenting symptoms. Only 30% of individuals with psoas abscess show the characteristic triad of fever, flank discomfort, and reduced range of motion in the hip joint [4]. In primary psoas abscesses, *Staphylococcus aureus* is the most prevalent causal organism and *E. coli* is rarely the causative organism in secondary psoas abscesses. The treatment of a psoas abscess is dependent on both the patient and the condition. Pre-existing medical issues and surgical readiness are two factors that patients must consider. The size of the iliopsoas abscess and the pathogenic organisms are both factors in the disease. Drainage and prompt initiation of adequate antibiotic therapy are the mainstays of iliopsoas abscess treatment. The majority of minor abscesses can be treated with antibiotics alone. Those who need drainage, however, can benefit from imaging studies like USG-guided or CT-guided drainage. Antibiotic treatment should be adjusted based on identified causative organisms for a minimum of 14 days [5].

The psoas muscle is in close relationship with all the major abdominal and pelvic structures, and any infectious process in these regions can spread to the psoas muscle and progress into the posterior mediastinum or anterior thigh. Secondary psoas abscesses, unlike primary abscesses, occur in older and more debilitated people with pre-existing diseases like CKD, as in our case. Our patient had no complaint of burning micturition but urine culture showed the growth of *E. coli.* In this patient, the possible source of *E. coli* was a renal cause, i.e., urinary tract infection and other causes of skeletal origin and in the alimentary tract.

Antibiotics are used to treat the abscess, as well as the draining of the abscess. The initial selection of antibiotics should be guided by a thorough understanding of the causative organisms but empirical antibiotic therapy should be started, which covers Gram-positive plus methicillin-resistant *Staphylococcus aureus* (MRSA) plus Gram-negative and anaerobic organisms. Adjustments should be made based on the abscess fluid culture and sensitivity results. Anti-staphylococcal antibiotics should be administered before the culture findings in patients suspected of having a primary psoas abscess. It is always best to start the patient on broad-spectrum antibiotics like clindamycin, anti-staphylococcal penicillin, and an aminoglycoside when they have a subsequent psoas abscess [6].

Patients with iliopsoas abscesses typically suffer from limited hip movement, and they prefer to be in hip flexion and lumbar lordosis position. When executing activities that stretch the psoas muscle, the pain is intensified. The pain caused by hip extension is known as the "psoas sign." The "psoas sign," combined with decreased hip discomfort during hip flexion, may help the clinician diagnose the illness.

## Conclusions

A psoas abscess should be considered as an alternative diagnosis in patients with leg pain, fever, and antalgic gait with reduced hip movement. In order to promptly recognize iliopsoas abscess, treating physicians should be alert to the various clinical presentations. To lower the risk of complications like septic shock and to achieve favorable outcomes, early imaging should be considered.
